# Confederate States Hospital Reports

**Published:** 1864-03

**Authors:** 


### CONFEDERATE STATES HOSPITAL REPORTS.

*Eighteen Cases of Gun-shot Wounds of the Head, observed at the General Hospital, Charlottesville, Va.*

Surgeon J. L. Cabell, in charge.

Case 1.—J. W. Hambleton, private Latham's Virginia battery, aged 37, entered the hospital August 11th, 1862, with a gun-shot wound, received August 9th, at the battle of Cedar Run, in the left temple, at or near the junction of the temporal with the parietal bone. The skull was found broken and pieces depressed. There was no paralysis and no mental aberration, but the night before the operation, he was possessed with the idea that he would die in a very short time. He was operated on August 24th. In this case the trephine was used, and all the broken pieces were removed. About a week after the operation, the patient had a slight attack of erysipelas of the scalp, but soon recovered from it. He has entirely recovered, but is injuriously affected by the heat of the sun.

Case 2.—John Cotton, private 17th Georgia regiment, company "G," aged 23, received a gun-shot wound August 9th, 1862, at Cedar Run, on the left side of the head, near the parietal protuberance—fracturing the bone, the internal table more extensively than the external, (this, I believe, is generally the case.) The mind was unaffected, but there was slight paralysis of the right side of the body. He was operated on September 14th—it was found necessary to use the trephine. Several pieces of bone were removed. Ten days after the operation, erysipelas supervened, but was readily subdued. This case did well, constantly improving, until Oct. 9th, when he was furloughed, at which time his wound had healed. He has not since been heard from.

Case 3.—W. C. Allen, private 1st Georgia regiment, company "E," aged 22, admitted Aug. 27th, 1862, with a gun-shot wound of the head. His general health was good. The wound in the scalp was about an inch and a half above the zygomatic process, and half an inch anterior to the left ear, *i. e.* a vertical line drawn along the front of the ear. The wound was suppurating slightly, but there were no symptoms indicating any serious injury to the skull or brain. The patient was going about as if nothing was the matter, and eating heartily. When admitted, the probe could not be introduced to the skull—the track of the wound being closed, probably by a firm clot, which had not been discharged by suppurative action. At the next examination with the probe, four or five days after his admission, the probe readily passed downwards under the temporal muscle, to the bone, which was fractured and depressed, but to what extent, could not be ascertained except by cutting. The seat of fracture was about three-fourths of an inch above the zygoma, and to reach it, required division of the swollen and puffy integument, and the temporal muscles in the vicinity of the fracture. A consultation was held, and an operation determined on. The ball, a common musket one, had been removed through the wound by a surgeon, shortly after the injury was received.

The operation was performed September 2nd, one week after his admission, as follows: I cut through the scalp and muscle beneath, making two incisions, one vertical, the other horizontal, crossing each other at the wound in the scalp, and making each about two inches in length. In doing so, it was necessary to apply two ligatures to the temporal artery and a branch of it. When the different flaps were partially dissected up, the fracture was found to be quite irregular, and as large, or larger, than a twenty-five cent piece, while the fragments were driven in and pressing upon the dura-mater. With considerable difficulty, thirteen pieces of bone were removed with the forceps, several of them were quite large and grooved on the inner surface, showing the seat of injury to be directly over the middle meningeal artery. After removing all the pieces that could be felt with the finger, the parts were drawn together by strips of adhesive plaster, and the wound dressed with wet lint. The patient had been put under the influence of chloroform, and kept so, during the operation, but it required three or four assistants to hold him, the chloroform merely depriving him of sensibility. The next day slight erysipelas had made its appearance around the wound, involving the ear and side of the face, and nearly closing the left eye. He was directed to take ten drops of muriated tincture of iron, every two hours, and to keep perfectly quiet. In twenty-four hours the erysipelas had nearly disappeared and the iron was discontinued during the day.

From the third day after the operation, no unpleasant symptoms occurred, the wound suppurating finely and rapidly closing; and this too with the patient going about the hospital more or less every day, as it was found impossible to keep him in bed. The treatment after the disappearance of the erysipelas, consisted solely in the application of wet lint twice a day to the wound, and keeping it clean.

September 18th.—The wound has healed, except just at the intersection of the two incisions. A small opening, the size of a probe, exists at this point, through which a slight discharge is kept up.

Case 4.—M. V. Jennings, private 35th Georgia regiment, company "B," aged 18, received a gun-shot wound in the head, near the parietal protuberance, making a slight groove or depression in the bone. The wound was received August 9th. When he entered the hospital he had jaundice. The side opposite to the wound was paralysed. No regular record was kept of this case. He was not operated on. Furloughed October 7th, with slight improvement.

Case 5.—Benj. Bird, private 16th Virginia regiment, company "F," aged 30, received a gun-shot wound August 30th, 1862, in right temporal region, fracturing the bone and depressing the fragments. He was operated on September 6th, and the pieces removed. The wound healed in six weeks without a single unfavorable symptom.

Case 6.—Isaac Souls, private 23d South Carolina regiment, company "H," aged 19, wounded August 30th, 1862, in the forehead, near the anterior fontanelle, and directly on the middle line. His general health was good. He was operated on September 5th, and the trephine was used. The saw was applied to the right of the middle line to avoid the longitudinal sinus; but, curiously enough, it happened to be one of the cases in which there was a lateral deviation of that sinus, and when the disc of bone was removed, the sinus was found exposed, but uninjured, as far as could be seen. One week after the operation, severe chills set in, followed by fever; then double pneumonia, of which the chills were precursors, made its appearance, with symptoms of pyæmia; and sixteen or seventeen days after the operation, the patient died suddenly during a fit of coughing, from rupture of the longitudinal sinus and profuse hemorrhage. *Post-mortem* revealed ulceration of the coats of sinus, with small spiculæ of bone resting upon them.

Case 7.—William Robertson, private — Alabama regiment, company "E," aged 26, wounded September 17th, 1862, on the right side of the forehead, at the root of the hair and below. The bone was extensively fractured and depressed, at or near the temporal ridge. No paralysis existed, and but little pain. He was operated on October 10th, at which time he had become much enfeebled. The scalp was undermined and the bone denuded around the fractured portion, and large quantities of pus were discharged daily. During the operation, it was found necessary to use Hay's saw, to release a large fragment of depressed bone. No unpleasant symptoms followed, and the patient finally recovered with a large and depressed cicatrix.

Case 8.—G. H. Sandford, private 8th Georgia regiment, company "A," aged 27, wounded August 28th, 1862, in the head, fracturing both tables of the superior portion of the left parietal bone, and depressing them. In this case, there was paralysis of the right side of the body, but the mind was clear. He was operated on September 23d, and all the pieces removed. The paralysis was entirely gone ten days after the operation. The patient rapidly improved and was furloughed and sent home soon after. He is believed to have recovered entirely.

Case 9.—B. W. Wells, corporal 3d South Carolina regiment, company "F," aged 24, wounded December 13th, 1862, "two inches above and to the left of the occipital protuberance." The wound in the scalp was three inches in length, and the bone was denuded for two and a half inches and slightly fractured. There was no depression that could be detected. This case was not operated upon. He seemed to be doing well for three or four days, then became restless; he had no fever, but soon became delirious. December 20th, coma supervened; afterwards, spasms—at first, of facial muscles, then it became general, and on the 21st he died.

Autopsy showed the external table slightly fractured, and the internal, also, for about an inch, but nearer to the middle line of the skull. About one ounce of extravasated blood was found between the skull and dura-mater, and a like quantity between the latter and the brain. The brain itself was disorganized to the depth of three-fourths of an inch and to the extent of about two square inches.

Case 10.—J. W. Abernethy, sergeant 15th South Carolina regiment, company "B," aged 26, wounded in the head December 13th, 1862, four inches above the right ear, fracturing the skull.

December 16th, 2 o'clock, P. M.—Wound clear. Paralysis of left arm and leg. Tongue protruded straight. Temperature of the left side lowered. Cerebal matter oozing from the wound. Dr. Davis operated, making a Y incision, removing a minnie ball, the base of which was one-third of an inch below the level of the internal tube; and a piece of the skull one inch in diameter, which it had carried before it, with a much smaller piece at the same level. Operation performed under chloroform. After removal of foreign bodies there was quite free arterial hemorrhage from the bottom of the wound, which subsided in about fifteen minutes; but some oozing continuing, per sulphate of iron was applied for two or three minutes, on a dossil of lint.

December 18th.—Pulse 100–2. Slept well on the night of the operation from solution morphia two drachms. Took none last night and slept badly, (owing he says to being cold.) Is, and has been, rational. Appetite very good. No stool since the operation. A large piece of bone came from the wound this morning. He complains of the left arm and leg feeling asleep, and aching in the knee and elbow. Tongue moist and clean. No hemorrhage from the wound. A clot was removed yesterday. Discharge free and sanious.

December 19th.—complained at dark last night of insatiable thirst, then had convulsions. Vomited and became comatose; at the same time, considerable hemorrhage occurred from his wound. He now passes his urine involuntarily. The right arm is forcibly flexed. The left and both lower extremities are relaxed. In this condition he sank rapidly, and expired at six P. M. No autopsy was made.

Case 11.—J. T. Damron, private 18th South Carolina regiment, company "H," wounded in the forehead, about two inches above the centre of the left eye. Date of the wound not remembered. He entered the South Carolina hospital September 6th, 1862, a few days only after its reception. He was operated on and several pieces of bone were removed. There were no *head* symptoms, and no paralysis existed. The case did well and he was furloughed October 8th, nearly well.

Case 12.—E. Pellum, private Holcombe Legion, ———, aged ——, received a gun-shot wound just above the right eye, fracturing the bone about the middle of the supra-orbital arch. The fracture extended upward a short distance along the frontal bone, and backward along the orbital plate. He entered the South Carolina hospital September 6th, a few days after the reception of the injury, and was operated on the same day, as case 11th. The loose fragments were removed, including portions of the orbital plate. No bad symptoms ensued and he was furloughed October 8th, nearly well.

Case 13.—A. McDonald, private Palmetto Sharpshooters, ———, aged ——, wounded in the forehead at the outer extremity of the left frontal sinus, fracturing the bone to a considerable extent. His wound was received a few days prior to the 6th of September, 1862, at which time he entered the South Carolina hospital. He was operated on the same day, with cases 11 and 12 The outer wall of the sinus was removed; the inner was found uninjured. This man was furloughed Nov. 11th, nearly well.

Case 14.—S. D. M. La Coste, sergeant 23rd South Carolina regiment, company "K." This was the fourth case occuring in the South Carolina hospital in this place. He was wounded, and received here at the same time with cases No. 11, 12 and 13. His wound, also, was in the forehead, a little to the left of the middle line, midway between the eye and the root of the hair. There was an indentation of the bone, but no perceptible fracture. The periosteum was gone to the extent of about one square inch.

An operation in this case was not considered justifiable. The man at this time, July 1st, 1863, (I am informed,) is at home, not yet recovered, and suffering constantly with intense pain in the head, regretting that he was not operated upon.

Case 15.—The following case was not seen by a hospital surgeon until a few minutes after his death, as he was staying at a private house and was attended by a private physician:

G. A. Harrell, lieutenant-colonel 14th Tennessee regiment, aged 55, wounded at Cedar Run, August 9th, 1862. He was struck by a ball about midway between the mastoid process and the vertex on the left side. A single piece of bone was driven in upon the dura-mater, about the size of a twelve and a half cent piece, but irregular in outline. It was removed after death with forceps. He was delirious most of the time, and for some hours before his death, comatose. His death occurred August 14th.

Case 16.—N. M. Morris, major 14th Tennessee regiment, aged 30, wounded at the second battle of Manassas, August 30th, 1862. A common musket ball struck the left side of the head, a little in front of, and on a level with the parietal protuberance. The skull was broken for about two and a half inches longitudinally, (*i. e.* horizontally) and about one inch across. The wound in the scalp was still larger. The ball remained in the wound. He was not examined by a surgeon on the field, as no one who saw him thought he would live beyond a few hours at the most. The day following, a large piece of bone was removed by his brother, with his finger. He was perfectly unconscious for five days, and when, at the expiration of that time, consciousness returned, he was found to be paralysed on the right side of the body. Three months after the reception of the wound, the ball was removed, as also a large piece of bone. Several smaller pieces were taken away, or discharged, at intervals; the last of them about four months after he was wounded. His brother says that some of the brain came from the wound shortly after the wounding, so that the membranes were probably extensively lacerated. His general health had not been good for four months before he was wounded, having suffered from chronic diarrhœa; this ceased, however, at the time. At present, July 1st, 1863, he is walking about with the assistance of a crutch. His paralysis is getting very slowly better; but he speaks with a good deal of difficulty. He frequently forgets what he is talking about, and says he cannot read anything from inability to connect the words into a sentence. His appetite is good and general health much improved. The wound is not yet entirely healed, and probably will not be for some time to come. There is a large cicatrized surface covering a depression two and a half inches long and three-fourths of an inch wide, beneath which, when the head is bent forward, the pulsations of the brain can be seen and felt distinctly.

The foregoing account, I received from the major himself, never having seen him before it was written. I could not avoid coming to the conclusion at the time, that although his case certainly proved a man may recover from a terrible wound of the head, without active surgical interferance, it would have been far better for him, and saved him from a great deal of suffering, to say nothing of time gained, to have had the ball and fragments of bone removed at once, instead of waiting for them to become loose and come away spontaneously by a long and tedious process of suppuration. All the time running the risk of a disorganizing inflammation of the brain and its membranes, from their constant pressure and irritation.

Case 17.—S. M. Willingham, private 5th Alabama regiment, company "D," aged 25, wounded at Boonsborough, Maryland, September 14th, 1862, in the forehead, just at the root of the hair, and on the right side near the middle line. He was for a short time unconscious, and for several days after, was occasionally delirious. There was no paralysis. He was taken prisoner at the time and carried to Philadelphia, where he was operated on ten days afterwards, and the pieces removed without the use of chloroform. His wound has entirely healed, leaving a depressed cicatrix. He suffers occasionally with a severe headache, which he attributes to his injury.

Case 18.—T. Wilson, a prisoner, 5th United States cavalry, company "D," aged 28, wounded at Brandy Station, June 9th, 1863, in the head, on the left parietal protuberance, about four inches above the left ear. The man was insensible for several days, and partially paralysed on the right side. Four days after the reception of the wound, the ball and pieces of bone were removed. He is now (July 6th) apparently doing well and the wound healing. He never has any pain in the head, but complains of a constant numbness and pain in his right arm, with loss, to a considerable extent, of its motions; but this is rapidly improving. He bids fair to recover entirely.

*Remarks.*—The foregoing list of cases, drawn up by Surgeon Allen, from notes furnished by the different operators, exhibit results differing remarkably from those announced by Dr. McLeod, in his "Notes on the Surgery of the Crimean War," both as to the mortality of cases of gun-shot fracture of the cranium, and as to the dangers incident to the use of the trephine. There were but two cases of actual perforation of the skull by the ball, and one of these (case 16) has recovered with a gradually improving hemiplegia. The patient articulates well, walks without his crutch, and begins to use his arm. As to comminuted fracture without actual perforation by the ball, the foregoing record proves that, even under the moderately favorable circumstances of a public military hospital, gravity of prognosis is an exceptional phenomenon.

